# Supplementary material for: Pre-Pregnancy BMI Influences the Association of Dietary Quality and Gestational Weight Gain: The SECOST Study
Source: Int J Environ Res Public Health. 2019 Oct 4;16(19):3735. doi: 10.3390/ijerph16193735 (PMC6801791; doi:10.3390/ijerph16193735)
Supplement: Supplementary file 1 [file ijerph-16-03735-s001.pdf]

International Journal of
Environmental Research and Public Health

MDPI

Table S1. Component score and total score for Healthy Eating Index (HEI) for Malaysians among the respondents.

	**Possible range of score**	**Criteria for a minimum score of 0**	**Criteria for a maximum score of 10**
HEI Component			
Cereals and grains	0 to 10	0 or > 8 serving	6 – 8 servings
Vegetables	0 to 10	0 serving	3 servings
Fruits	0 to 10	0 or > 2 serving	2 servings
Milk and milk products	0 to 10	0 or > 3 serving	2 – 3 servings
Poultry, meat, and egg	0 to 10	0 or > 2 serving	1 – 2 servings
Fish and seafood	0 to 10	0 or > 1 serving	1 serving
Legumes	0 to 10	0 or > 1 serving	1 serving
% of energy from total fat	0 to 10	≥ 35% energy from fat	≤ 30% energy from fat
Sodium	0 to 10	≥ 4200 mg	≤ 2000 mg
Total HEI	0 to 100		

Note. If individuals less or exceed of the recommended servings, the score was calculated proportionately between 0 and 10. If an individual consumed less than the recommended amount of servings, the score was calculated with the following formula: 10×(the consumed amount of servings)/(the lower limit of the recommended serving). If an individual consumed more than the recommended amount of servings, the score was calculated with the following formula: 10−10×[(the consumed amount of servings)−(the upper limit of the recommended amount)]/(the upper limit of the recommended amount). Each score was rounded off to the nearest whole number. When this calculation produced a negative score because of excess servings, the score was converted to 0.
